# Prematurity and BPD: what general pediatricians should know

**DOI:** 10.1007/s00431-022-04797-x

**Published:** 2023-02-10

**Authors:** Luca Bonadies, Maria Elena Cavicchiolo, Elena Priante, Laura Moschino, Eugenio Baraldi

**Affiliations:** 1 Neonatal Intensive Care Unit, Department of Woman`s and Child`s Health, University Hospital of Padova, via N. Giustiniani 3, Padova, 35128 Italy; 2 Institute of Pediatric Research “Città della Speranza”, Padova, Italy

**Keywords:** Prematurity, Very low birth weight infants, Bronchopulmonary dysplasia, Follow-up, General pediatricians, Neonatology

## Abstract

More and more very low birth weight (VLBW) infants around the world survive nowadays, with consequently larger numbers of children developing prematurity-related morbidities, especially bronchopulmonary dysplasia (BPD). BPD is a multifactorial disease and its rising incidence in recent years means that general pediatricians are much more likely to encounter a child born extremely preterm, possibly with BPD, in their clinical practice. Short- and long-term sequelae in VLBW patients may affect not only pulmonary function (principally characterized by an obstructive pattern), but also other aspect including the neurological (neurodevelopmental and neuropsychiatric disorders), the sensorial (earing and visual impairment), the cardiological (systemic and pulmonary hypertension, reduced exercise tolerance and ischemic heart disease in adult age), nutritional (feeding difficulties and nutritional deficits), and auxological (extrauterine growth restriction). For the most premature infants at least, a multidisciplinary follow-up is warranted after discharge from the neonatal intensive care unit in order to optimize their respiratory and neurocognitive potential, and prevent respiratory infections, nutritional deficiencies or cardiovascular impairments.

*Conclusion*: The aim of this review is to summarize the main characteristics of preterm and BPD infants, providing the general pediatrician with practical information regarding these patients’ multidisciplinary complex follow-up. We explore the current evidence on respiratory outcomes and their management that actually does not have a definitive available option. We also discuss the available investigations, treatments, and strategies for prevention and prophylaxis to improve the non-respiratory outcomes and the quality of life for these children and their families, a critical aspect not always considered. This comprehensive approach, added to the increased needs of a VLBW subjects, is obviously related to very high health-related costs that should be beared in mind.

**Table:** 

**What is Known:** *• Every day, a general pediatrician is more likely to encounter a former very low birth weight infant.* *• Very low birth weight and prematurity are frequently related not only with worse respiratory outcomes, but also with neurological, sensorial, cardiovascular, renal, and nutritional issues.*	
**What is New:** *• This review provides to the general pediatrician a comprehensive approach for the follow-up of former premature very low birth weight children, with information to improve the quality of life of this special population.*

**What is Known:**

*• Every day, a general pediatrician is more likely to encounter a former very low birth weight infant.*

*• Very low birth weight and prematurity are frequently related not only with worse respiratory outcomes, but also with neurological, sensorial, cardiovascular, renal, and nutritional issues.*

**What is New:**

*• This review provides to the general pediatrician a comprehensive approach for the follow-up of former premature very low birth weight children, with information to improve the quality of life of this special population.*

## Epidemiology of prematurity and bronchopulmonary dysplasia

Preterm birth is a frequent event, happening in about 10% of pregnancies. Although very preterm and extremely preterm births occur less frequently nowadays, every year 1.1% of newborn infants are born at less than 28 weeks of post-menstrual age (PMA), and with a birth weight of less than 1500 g, defined as very low birth weight (VLBW) [1].

Recent advances in neonatal care have enabled more infants to survive extremely preterm birth, giving rise to more children with prematurity-related morbidities like bronchopulmonary dysplasia (BPD). However, efforts to minimize lung injury have not reduced the incidence of BPD [2], so general pediatricians are increasingly likely to encounter preterm-born children with or without BPD in their clinical practice.

BPD is diagnosed most commonly as oxygen need for 28 days after birth and graded with respiratory support at 36 weeks PMA [3–5]. Depending on how BPD is defined, it affects from 6 to 57% [6] of preterm infants. The most often-used definitions of BPD [3–5] have been developed to help us to classify infants and to predict the long-term respiratory outcomes of BPD subjects [7].

Even VLBW infants not diagnosed with BPD can experience some degree of lung abnormality during childhood and adulthood, suggesting the presence of a chronic lung disease of prematurity. Their poor lung function poses a risk of a chronic obstructive pulmonary disease (COPD)-like phenotype later in life. Hence the growing attention to monitoring lung function and managing respiratory symptoms in VLBW subjects beyond the neonatal period.

## Pathogenesis of BPD

BPD is a multifactorial disease [8] thought to develop already in utero as suggested by metabolomics studies on amniotic fluid [9]. This is part of a wider pathogenic picture in which pre- and post-natal factors affect the immature lungs, making them highly vulnerable to inflammation, direct injury, and impaired development, possibly predisposing them to BPD [10]. The noxae involved include intra- and extra-uterine growth restriction, preeclampsia, chorioamnionitis, maternal smoking, hyperoxia, mechanical ventilation, sepsis, hemodynamically significant patent ductus arteriosus (PDA), and microbiota, all factors that can disrupt lung development. Associated with aberrant repair mechanisms, these factors can exacerbate the histopathology of the lung. BPD is characterized by a reduced alveolar surface area available for gas exchange, an altered angiogenesis with possible consequent pulmonary arterial hypertension, and a widening and thickening of the interstitial spaces due to fibrotic repair mechanisms [8]. Reasonably, the extremely vulnerable lung of the smallest preterm is unlikely to reach term equivalent age without any sign of lung disease, even if not classified as BPD, again suggesting the presence of a chronic lung disease of prematurity [11], in which those with BPD are likely to be the more severe group.

## Prevention and early treatment of chronic lung disease during the neonatal period

Several interventions have shown potential for acting on the above-mentioned factors and limiting the burden of BPD and respiratory sequelae of prematurity. They include [12] antenatal corticosteroids; early surfactant administration with less invasive techniques; gentle and protective mechanical ventilation (when necessary); targeted oxygen saturations; early caffeine therapy; postnatal infection control; treatment for hemodynamically significant PDA; and adequate enteral nutrition, preferably with human milk.

There are few options for the pharmacological treatment of evolving BPD and BPD during the newborn’s hospital stay. Systemic corticosteroids are among the most studied: they have shown promise in reducing the incidence of BPD, especially when used within the first 8–14 days of life [13]. The risk of side effects, such as worse neurodevelopmental outcomes [14] and higher mortality rates [15], raises questions over their use, however, and there is still no consensus on the best choice of drug, its dosage, and the timing of its administration [16].

Other frequently prescribed drugs are diuretics, like furosemide, chlorothiazide, and spironolactone, which may be started in hospital, then discontinued at home, depending on the infant’s need for oxygen therapy and post-discharge growth [17].

Preclinical and early clinical studies have generated positive results with stem cells [18, 19] and their products (extracellular vesicles) [20], raising the need for early biomarkers of respiratory outcomes to identify patients at high risk and treat them early with these potentially breakthrough therapies [21, 22].

Meanwhile, general pediatricians will see increasing numbers of preterm-born children with chronic lung disease persisting into adult life [10].

To better support this population, general pediatricians and neonatologists need to work together even before patients are discharged from the neonatal intensive care unit (NICU). Pediatricians should meet the infants and their parents already in hospital and plan the patients’ short-term clinical follow-up with the neonatologist. This is especially crucial for the most fragile infants, like those needing oxygen supplementation at home.

## Respiratory outcomes and symptoms after the neonatal period

Children born extremely preterm are at higher risk of both short- and long-term respiratory sequelae [23]. Among them, those with BPD stay longer in the NICU, and are sometimes discharged home with oxygen supplementation [24]. Tracheobronchomalacia is another complication of prematurity and BPD, and a risk factor for longer hospital stays and respiratory problems [25]. The affected infants will consequently need closer monitoring at home.

During the first 2 years of life, infants born extremely preterm, with or without BPD, are hospitalized more often than children born at term [26, 27], suggesting that this population frequently has some degree of lung impairment [11]. These infants also have an immature immune system [28], which puts them at higher risk of severe respiratory infections that may require hospitalization and intensive care, especially in presence of BPD-related pulmonary hypertension [29]. The main pathogens involved are respiratory syncytial virus (RSV) and rhinoviruses that can make preterm subjects’ fragile respiratory systems reach the symptomatic threshold more easily. Exposed to repeated infections and respiratory re-exacerbations, the patients’ already impaired lung structure probably worsens, giving rise to frequent coughing and recurrent wheezing episodes. Pre-school and school-aged children with BPD are consequently at higher risk of asthma-like symptoms.

Monitoring lung function in preschoolers born preterm is not an easy task. It entails using non-routine methods like the forced oscillation or interrupter resistance techniques. Such methods have demonstrated reduced expiratory flows in preterm infants regardless of any BPD diagnosis [30], but results are worse in infants known to have BPD [31]. Lung function has also been found to correlate better with respiratory symptoms than severity of BPD [32].

Several children with airway flow limitations (reduced FEV1) in school age do not improve as they grow older [33, 34]. In adulthood, thanks to a catch-up alveolarization through childhood, they might have alveolar dimensions and number comparable with those of healthy 10- to 14-year-olds, despite a significant difference in FEV1 [35]. The dysanaptic concept may explain this “non-parallel” growth in lung size and airway caliber, such that individuals with BPD and term-born controls differ in expiratory flow rates despite similar lung sizes. It is not clear when and how their flow limitations occur and evolve, but several studies show that individuals born significantly preterm do not match their peers’ lung function at 20–25 years of age, and consequently carry a higher risk of early COPD symptoms [10, 36–38]. Infants with intra-uterine growth restriction (IUGR), as well as being at increased risk for BPD, have an even worse respiratory function when compared to non-IUGR infants at a mean age of 11 years [39, 40].

Children born very preterm, and those with BPD especially, may have an impaired exercise tolerance that persists in adolescence and adulthood [23]. This could be due to multiple alterations in their cardiorespiratory system, including airway flow limitation, reduced pulmonary vascular capacity, lower peak oxygen consumption and anaerobic threshold, and altered respiratory mechanics [41].

## Treatments available beyond the neonatal period

A main concern for general pediatricians is the current lack of treatments for symptoms of chronic lung disease and its re-exacerbations. Drugs for asthma are frequently prescribed to former BPD and preterm subjects, especially inhaled corticosteroids and bronchodilators [42], but this choice is not based on scientific evidence.

The chronic inflammation in BPD subjects [43, 44], and the normal levels of FeNO (a marker of eosinophilic inflammation) in children born preterm suggest that the inflammatory mechanisms in their airways differ from the eosinophilic inflammation of childhood asthma, raising questions over the long-term use of inhaled corticosteroids for BPD patients [45]. Research on the efficacy of these drugs in preterm-born children is still ongoing [46], but there is still no evidence to support their background use [47]. An expert consensus of the European Respiratory Society (ERS) [42] recommended against using inhaled corticosteroids (low certainty of evidence), and stated that their effects should be carefully monitored during a trial period before any long-term treatment.

Spirometry has documented a certain response to bronchodilators in BPD subjects [48], but the clinical significance of this amelioration has yet to be demonstrated. Chronic use of short-acting bronchodilators (salbutamol, fenoterol) is therefore not recommended, given the adverse effects associated with their overuse as a single therapy described in asthmatic patients.

The ERS task force [42] suggests bronchodilators only for specific subgroups of children with BPD: the most severe cases; those with asthma-like symptoms; those repeatedly hospitalized due to respiratory morbidity; and those with exercise intolerance or evidence of reversible airway obstruction.

Summing up, the evidence on chronic and acute therapies for BPD and its re-exacerbations is scarce. Treatments should be adequately tailored to individual patients. In cases of moderate-severe BPD, or frequent re-exacerbations, we recommend follow-up visits with pediatric pulmonologists, who can help general pediatricians assess patients’ respiratory symptoms and monitor the benefits of any therapies, preferably measuring lung function [49].

On the matter of preventive measures, the picture changes completely.

Palivizumab is a monoclonal IgG antibody against the RSV’s protein F. Adopting a monthly administration protocol, it has been demonstrated to reduce (55%) the hospitalizations for severe RSV infections involving preterm-born infants, especially those with BPD. There is still no standardized European guideline on the use of palivizumab, and national guidelines follow cost-effectiveness considerations [50]. In general, prophylaxis should be considered during the RSV season for infants with BPD in their first year of life. In their second year, it is recommended for infants who continue to require respiratory support (chronic corticosteroid and bronchodilators therapy, diuretics or supplemental) during the 6-month before the start of the RSV season [51, 52]. Palivizumab is considered safe and effective, but the need for repeated injections can be a problem. This will be overcomed by a new single-shot monoclonal antibody, Nirsevimab, with a 5 months half-life [53–55].

Given the immature and less competent immune system of infants born preterm, their immunization schedules should not be delayed. Vaccinations should be administered at the same chronological age and according to the same schedule as for term-born infants. Exceptions include: the hepatitis B birth dose, which should be deferred until discharge from hospital or one month of age for infants born at < 2000 g from mothers who test negative for the hepatitis B surface antigen; and the rotavirus vaccine, which is contraindicated in preterm-born infants who have had necrotizing enterocolitis [56]. Adverse events such as apnea can occur after immunization with vaccines [57], so the first dose may be administered in hospital, and before discharge whenever possible for high risk subjects [58, 59].

## Respiratory follow-up for children born very preterm and those with BPD

### Lung imaging

Using lung imaging to monitor the respiratory health of BPD subjects is not currently routine, but high-definition CT or MRI may be useful in cases of particularly severe respiratory symptoms and/or recurrent hospital admissions [42]. Despite the uncertain benefits of imaging and the radiation exposure risks, other diagnoses influencing such patients’ respiratory status need to be ruled out. A field worth exploring concerns lung ultrasound: it has proved valuable in the neonatal period [60], also for predicting BPD [21, 61], and has numerous applications in pediatric age [62, 63], but its use in the post-NICU follow-up subjects has never been explored.

### Lung function tests

There is no standard respiratory follow-up schedule for BPD, but a recent US experts’ survey recommended an early pulmonary consultation (1–2 months after discharge from the NICU) for infants with moderate or severe BPD, and at least a one-off specialist assessment for cases of mild disease [49]. Lung function evaluations in preschoolers former preterm could be performed only with non-routinary techniques, like forced oscillation or interrupter resistance techniques, but could be considered in the most severe cases [30–32]. Beyond 5 years of age, spirometry is an easy and non-invasive tool, so it is useful in the longitudinal follow-up of school-age children born preterm, especially if with BPD. Response to bronchodilators should be tested as well, and, if positive, pediatric pulmonologists should be consulted on the need for background therapy [64], and to monitor any effects.

### Oxygen therapy at home

General pediatricians have a crucial role in managing BPD infants discharged home with oxygen supplementation. The ERS consensus guidelines [42] recommend maintaining an oxygen saturation target of > 90%, but further studies are needed to establish optimal SpO2 targets in BPD infants. Supplementary oxygen at home for children with chronic neonatal lung disease aims to reduce or prevent pulmonary hypertension, contain intermittent desaturations, reduce airway resistance, promote growth and neurodevelopment, and possibly limit the associated risk of sudden unexplained death in infancy. Parents should be instructed on how to monitor and manage oxygen before their child is discharged, and reminded when at home. The use and evaluation of the pulse oximeter that is provided to oxygen dependent subjects could be trivial for a healthcare provider, but difficult and scary for the anxious parents, that can over- or under-estimate the presence of an alarm. Parametric thresholds should consequently be adequately set and their meaning explained to the caregivers. Parents will also need guidance on the gradual weaning of oxygen supplementation when the time comes [65].

### Daycare center attendance

Parents of children born preterm will likely ask for their general pediatricians’ advice about attending daycare centers or kindergarten. Despite the limited scientific evidence available, this advice should take into account local experience, the child’s age, the time of year, the parents’ wishes and possibilities. The first winter of very young infants with severe BPD is clearly of greatest concern, but many factors could influence parents’ decisions, and the potential benefit of kindergarten to their child’s social development should not be overlooked [42].

### Smoke avoidance and Hygiene

Caring for children born preterm also includes: avoiding second- and third-hand smoking and the related preventable inflammation and oxidative stress of their already-damaged lungs, and to protect their respiratory health [66]; avoiding crowded places, and rooms full of older children; and ensuring adequate hand hygiene of the care-givers [67].

## Other areas of intervention

Prematurity affects multiple systems with potentially lifelong consequences [68], Crump et al. have suggested that “preterm birth should be recognized as a chronic condition that requires long-term follow-up” [69]. The European Foundation for the Care of Newborn Infants (EFCNI) has developed standards of care for infants born preterm from birth to school age. They propose a holistic approach, covering respiratory, cardiovascular and neurological aspects, but also promoting healthy habits, mental health and peer relationships [70]. This would optimize numerous outcomes, and particularly the child’s neurocognitive potential, through the early identification of motor, social or cognitive issues and the provision of appropriate rehabilitation.

### Neurological outcomes

BPD is independently associated with adverse neurological outcomes at 12 and 24 months of corrected age. On Bayley III assessments, impairments in one or more neural functions relating to adaptability, gross motor, fine motor, language, or social skills are significantly more common in preterm-born children with BPD. Pulmonary hypertension can also have a negative influence on such neurodevelopmental outcomes [71].

Recent brain MRI studies comparing term-born infants with those born preterm and developing BPD showed a delayed brain maturation in the latter [72]. BPD also influenced cerebellar development [72]. Despite this evidence, brain MRI is suggested for VLBW infants at term equivalent age [73], but after discharge is not a routine part of the BPD infant’s follow-up, when other neurological issues are not suspected.

IUGR is a worsening factor also in the neurological development, being associated with both alterations in brain growth on MRI evaluation at term and poorer toddlerhood outcomes. Infants with a previous diagnosis of IUGR present lower cognitive and motor scores at 22 months and a positive Modified-Checklist for Autism (OR 2.12) compared with adequately grown controls [74].

Infants born preterm are also at higher risk of neuropsychiatric disorders during childhood. The “preterm behavioral phenotype” features a higher risk of symptoms and disorders associated with inattention, anxiety and social difficulties, and a markedly greater prevalence of autism spectrum disorders [75], but also psychosis in adult age [76]. General pediatricians suspecting such issues should refer patients for specialist assessment at an early stage.

Finally, preterm-born infants with severe brain lesions such as stage 3 or 4 intraventricular hemorrhage, periventricular leukomalacia or a history of altered findings on electroencephalography (EEG) should be monitored with serial EEG because they are at higher risk of developing epilepsy [77].

### Feeding difficulties

Premature and BPD infants may experience feeding problems, and swallowing dysfunctions that can affect their growth and respiratory function. BPD infants may have tachypnea or low oxygen saturation episodes during breastfeeding due to a poor coordination of sucking and swallowing, and gastroesophageal reflux is common [78]. Little is known about suitable nutritional interventions [79]. Human milk is the best choice also for this population, with the possibility of caloric and proteic supplementation. If a human milk fortifier is used for this purpose, it could be continued up to a corrected age of 56 weeks [80]. When human milk or breastfeeding is not enough to cover the baby’s request, a post-discharge formula rich in calories (72 kcal/100 ml) or a preterm formula (80 kcal/100 ml) may be continued in VLBW infants with extrauterine growth restriction.

Feeding problems, diuretics, postnatal steroids, and an inadequate intake of minerals may impair optimal growth in the first years of life, with consequent deficiencies (such as low iron levels or metabolic bone disease) in preterm infants [81]. So, supplementing vitamins and other elements is mandatory for preterm infants.

Vitamin D contributes to bone mineralization and neuromuscular function, and can also have an anti-inflammatory function [82]. The European Society for Pediatric Gastroenterology, Hepatology and Nutrition suggests a dose of 800–1000 IU/day till full-term then 400 IU, whether the premature infants are fed mother’s milk or formula [83]. Iron is essential for neurodevelopmental outcomes of VLBW infants. Supplementation with a 2–3 mg/kg oral dose of iron is recommended at least until 6–12 months old, depending on the introduction of complementary feeding. A recent meta-analysis on enteral calcium or phosphorus supplementation in preterm-born infants found insufficient evidence of any improvement in the children’s growth and bone health [84]. General pediatricians should assess feeding and nutrition regularly, taking anthropometric measurements and asking parents about any respiratory or digestive problems. A pediatric gastroenterologist and should follow-up infants with gastrointestinal problems requiring the use of a nasogastric tube or a transcutaneous gastrostomy and a nutritionist/dietician could help improve caloric intake.

### Auxological parameters

Most VLBW infants are discharged home without an optimal growth during their hospital stay, when the targeted caloric intake is not always achievable [85]. The resulting extrauterine growth restriction means that infants’ auxological parameters are often below the 10th centile at the time of their discharge, but they tend to catch up afterwards. They usually reach adequate growth parameters over the first two years (but can sometimes take 5–6 years) [86], and a better growth seems related to a better neurodevelopment [87].

### Cardiovascular and nephrological outcomes

Cardiovascular problems associated with prematurity, such as pulmonary hypertension, need monitoring with echocardiography and specialist cardiologist consultations. Young adults born preterm are also at higher risk of non-communicable diseases [88] like ischemic heart disease [89], hypertension [90], high fasting glucose and total cholesterol levels [91]. It is therefore wise to check premature-born children’s arterial blood pressure during visits to the general pediatrician, and to schedule echocardiography for selected high risk preterm infants, when they reach school age. Multiple earlier assessments are recommended for those still on supplemental oxygen therapy or with abnormal findings at the time of discharge from hospital [64]. Again IUGR subjects seem at higher risk for cardiovascular morbidity and mortality, not only as a consequence of the metabolic diseases, but for the evidenced alterations of cardiac and vasculature structure and function [92].

The numerous aspects of metabolic syndrome (insulin resistance, obesity, and dyslipidemia) should be clinically monitored too, especially in VLBW infants with a history of IUGR, which are at higher risk [93], and it is important to highlight this risk when pediatricians hand over their patients to general practitioners. In fact, individuals born very preterm are also at greater risk of chronic kidney disease (as 60% of nephrons develop in the last trimester of pregnancy) from childhood into mid-adulthood [94], and this adds to their risk of systemic hypertensive disease.

### Physical activity

Assessing children’s physical activity levels should be part of the general pediatrician’s routine, especially for children born preterm. Physical activity is recommended in this population, given its known benefits [95], which could positively influence some cardiovascular and metabolic aspects. One study found that even a short (4-week) exercise program could significantly improve the results of the 6-min walking test, incremental shuttle walk test, modified sit and reach test and spirometry in 5-year-olds born preterm [96]. In another study, significant differences in some baseline cardiac MRI parameters between adults born preterm and term-born controls disappeared after the attendance of 14 weeks of thrice-weekly supervised aerobic exercise and resistance training [97].

### Audiological and ophthalmological assessments

It is strongly advisable to test preterm-born infants’ sensory functions because their early exposure to numerous drugs (especially antibiotics and loop diuretics) can cause hearing impairment. To identify and treat this issue early on, screening is usually done before discharge, but a follow-up visit should be scheduled a few months later to check infants’ hearing function and ensure any necessary action is taken before they are 6 months old [98].

Visual function should be monitored too, as preterm infants are more likely to have a reduced visual acuity, strabismus, abnormal stereopsis and refractive errors, but routine screening may not detect other problems, such as reduced visual fields, impaired contrast sensitivity, or deficits in cortical visual processing, that may occur in this population [99]. All these issues can interfere with the optimal development of infants born preterm, and their prompt identification and treatment can help to ameliorate the children’s quality of life.

### Family care

A final aspect that should be beared in mind concerns the preterm infants’ families. The birth of an extremely premature child dramatically upsets the balance of any couple and family. Then the infant’s discharge from hospital brings new disruptions, imposes new daily routines, adds to parents’ stress and anxiety, leaves them less time for their other children, and so on [100]. Identifying early red flags of parents’ difficulties or mental health issues can be crucial to the future of the whole family and to the infant’s development. Collaborating with national and international foundations like the EFCNI is extremely important to improving the long-term health and quality of life for preterm infants and their families [70].

### The “intensive care” team for the discharged VLBW subject

A comprehensive approach to the VLBW subject is consequently strongly recommended and will imply a large multi-specialistic team, coordinated by the general pediatrician and the neonatologist, but including pneumologists, cardiologists, neurologists, neuropsychiatrists, nutritionists, gastroenterologists, nephrologists, endocrinologists, radiologists, ophthalmologists, audiologists and speech, deglutition, and physical therapists. This should also include non-medical figures like support teachers, psychologists and social workers. The access to this panoply of services is not always easy and families could benefit from the support of patients’ associations that can help subjects and their families address the high demand for care and health needs associated with very preterm birth. Patients’ associations have also the important role of patients’ advocacy, speaking out their best interests and highlighting to every involved figure the importance of their role. One of these associations, the European Foundation for the Care of Newborn Infants (EFCNI), has fully accepted this responsibility, and recently published a comprehensive guideline (shared with numerous neonatal medical societies) for the care of all the facets of the newborn infant, especially if preterm born [70].

### Health-related costs for VLBW infants’ care

As can be deduced, a preterm born infant requiring a prolonged hospitalization with intensive care needs, implies quite high health-related costs. However, the first hospitalization is only the beginning of an expensive journey, requiring a significantly higher expenditure compared with the neonatal admission of a term born infant, as shown by many different papers on this topic showing similar results even if in different realities [101–103], with a particularly high cost when BPD is present [104]. The needs of VLBW infants obviously don’t end with the first hospitalization, but require many specialistic evaluations, medical and physical therapy, cognitive support and so on, implying increased health-related costs possibly through all their life [105], especially for those subjects with BPD [106]. A comprehensive approach to support all the possible critical aspects of the former VLBW subject, would probably imply a high expense at first, but could end in a better quality of life and possibly a lower level of needs subsequently. This aspect surely justifies the important initial disbursement, targeting the best potentiality of each VLBW infant.

To sum up, Table 1 lists the main follow-up assessments and interventions that general pediatricians should consider for former preterm patients, especially those with BPD.

**Table 1:** Follow-up evaluation and pharmacological intervention for VLBW infants during childhood

	**Evaluation**	**Intervention**
**Respiratory follow-up**	Forced oscillation technique (FOT) and interrupter technique (RINT) at preschool age when available Spirometry at school age (4–5 years) Dynamic airway imaging (CT or MRI) for severe cases	Palivizumab prophylaxis following national guidelines Suggestions for BPD infants: preterm infants < 32 GW + 0 with BPD in the first year of life; second year if respiratory medical support is required Vaccinations: immunization schedules following chronological age and according to the same national schedule as term infants Influenza vaccine during season in infants > 6 months Inhaled corticosteroids and bronchodilators can be considered based on clinical symptoms and spirometry results
**Neurological follow-up**	Evaluation of milestones and anthropometric measures Neonatologist clinical evaluation at 3–6–12 and 24 month corrected age Cognitive tests and scales (Bayley III or Griffith scale) at 12 and 24 months corrected age	Early rehabilitative intervention to optimize neuroplasticity and prevent complications Early logopedic support if language delay is suspected
**Cardiovascular and renal follow-up**	Echocardiography if persistence of pulmonary hypertension Electrolyte measurements in patients on diuretics Blood pressure measurements	For infants discharged from the NICU on chronic diuretic therapy: discontinuation in a judicious manner (weaned by allowing the dose to decrease slowly relative to the child’s weight) Pulmonary vasodilators: sildenafil with pediatric cardiologist support and monitoring
**Metabolic and hematological follow-up**	Glucose and total cholesterol levels monitoring Iron status, complete blood count with reticulocyte count, serum ferritin levels	Oral iron: 2–3 mg/kg/day of until 6–12 months of age (depending on the start of complementary feeding) Vitamin D: 400–800 IU once a day during the first year
**Gastrointestinal and nutritional/auxological follow-up**	Specialistic consultation if feeding problems Swallow evaluation if frequent cough or oxygen desaturation during feeding Weight monitoring Nutritionist evaluation if failure to thrive	Promoting breastfeeding/administration of human milk at discharge Swallowing physiotherapy Goal 120 kcal/kg/day: post discharge formula milk/preterm formula milk/human milk fortifier for extrauterine growth-restricted infants Individualized breastfeeding weaning and solid food introduction
**Special senses follow-up**	ROP and ophthalmologist visits Hearing follow-up	

## Conclusions

**Fig. 1:**
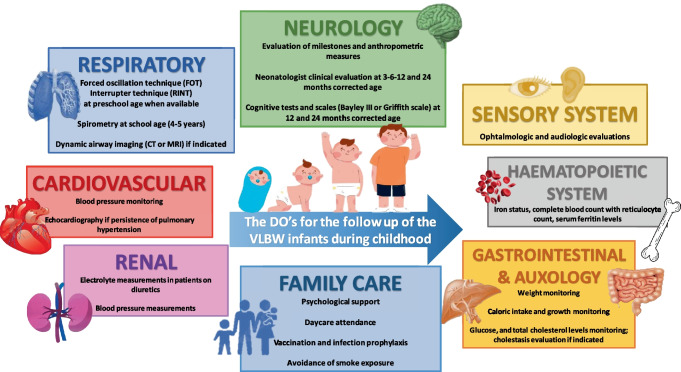
Graphically summarizes the main evaluations that needs to be performed during the follow-up of a former very low birth weight infant (the figure was realized with Powerpoint, Microsoft, USA)

VLBW infants with and without BPD warrant a long-term medical care that still has many aspects that remain unclear. After these infants’ discharge from the NICU, collaboration between the neonatologist and the general pediatrician is fundamental to the child’s proper follow-up and the discontinuation of previously-used therapies. Our increasing understanding of the main problems of children born very preterm suggests the need for a multispecialist follow-up into adulthood, especially for those with severe BPD [64] (Fig. 1). From pre-school age onwards, general pediatricians become the reference figure for preterm-born infants and their parents, so they need to be aware of the options available for improving the quality of life of this population.
